# Cellulose Nanofibers/Pectin/Pomegranate Extract Nanocomposite as Antibacterial and Antioxidant Films and Coating for Paper

**DOI:** 10.3390/polym14214605

**Published:** 2022-10-30

**Authors:** Enas Hassan, Shaimaa Fadel, Wafaa Abou-Elseoud, Marwa Mahmoud, Mohammad Hassan

**Affiliations:** 1 Cellulose and Paper Department, National Research Centre, 33 El-Buhouth Street, Dokki, Giza 12622, Egypt; 2 Advanced Materials and Nanotechnology Group, Centre of Excellence for Advanced Sciences, National Research Centre, 33 El-Buhouth Street, Dokki, Giza 12622, Egypt; 3 Food Technology Department, National Research Centre, 33 El-Buhouth Street, Dokki, Giza 12622, Egypt

**Keywords:** sugar beet pulp, cellulose nanofibers, pectin, pomegranate, nanoemulsion, antibacterial, antioxidant, papermaking, coating, migration

## Abstract

Bio-based polymer composites find increasing research and industrial interest in different areas of our life. In this study, cellulose nanofibers (CNFs) isolated from sugar beet pulp and nanoemulsion prepared from sugar beet pectin and pomegranate extract (PGE) were used for making films and used as coating with antioxidant and antimicrobial activities for paper. For Pectin/PGE nanoemulsion preparation, different ratios of PGE were mixed with pectin using ultrasonic treatment; the antibacterial properties were evaluated to choose the formula with the adequate antibacterial activity. The antioxidant activity of the nanoemulsion with the highest antimicrobial activity was also evaluated. The nanoemulsion with the optimum antibacterial activity was mixed with different ratios of CNFs. Mechanical, greaseproof, antioxidant activity, and antibacterial properties of the CNFs/Pectin/PGE films were evaluated. Finally, the CNFs/Pectin/PGE formulation with the highest antibacterial activity was tested as a coating material for paper. Mechanical, greaseproof, and air porosity properties, as well as water vapor permeability and migration of the coated layer from paper sheets in different media were evaluated. The results showed promising applicability of the CNFs/Pectin/PGE as films and coating material with antibacterial and antioxidant activities, as well as good stability for packaging aqueous, fatty, and acidic food products.

## 1. Introduction

Specialty paper products are important category of paper with wide commercial production and uses. Among these specialty paper products are those with antibacterial activity which find increasing use in packaging and hygienic products. Research in that area is progressing in increasing rates in order to reach economic, eco-friendly, and safe additives or chemical modification routes. Additives with antibacterial activity extracted from bio-based materials or non-food non-feed residues are of special interest since they have the benefits of being widely available, cheap, eco-friendly, and generally safe, e.g., having no health hazard. Among these residues is pomegranate peels, which are rich in antibacterial and antioxidants extractives, Hundreds of tons of pomegranate peels are available every year at food companies all over the world. Pomegranate peel extractives (PGE) are widely studied and used in food, pharmaceutical, and medicinal applications due to their powerful antibacterial, antifungal, virucidal, and antiviral activity [1,2,3,4]. PGE is rich in many antioxidant, antibacterial, and antiproliferative compounds [5]. To improve the solubility of PGE and its application in aqueous solutions, their use in forms of emulsions is considered a good practice. Ethanol-extracted PGE can be formulated into nano-size emulsions using proper emulsifying agents [6,7,8,9,10]. The nano-size emulsions have the privilege of a very high surface area, and thus could be used effectively in smaller amounts than the micro-emulsions. Emulsifying agents can be of synthetic origin such as polyglycerol polyricinoleate and Tween 80, which are used for the preparation of nanoemulsion from pomegranate extract [8]. Nevertheless, the use of bio-based surfactants are preferred when it comes to food, food packaging, or cosmetic products. For this reason, maltodextrin and whey protein isolate [6,10], sodium carboxymethyl cellulose [7,11], and chitosan [9] have been used for preparation of PGE emulsions. In this aspect, sugar beet pectin in particular has high emulsifying activity when compared to pectin isolated from other food residues, such as citrus and apple peels, due to its specific chemical composition, the presence of a high degree of acetylation, and low molecular weight [11]. On the other hand, pectin from other resources, such as citrus and apple peels, have poor emulsifying properties but have other features such as thickening, gelling, and film forming properties thanks to their much higher molecular weight and much lower degree of esterification than that of SBP [12,13].

It is worth mentioning here that sugar beet pulp (SBP) is a common agricultural residue in different areas of the world, as 20% of the world production of sugar comes from sugar beet [14]. After sugar extraction, SBP residue is rich cellulose, hemicellulloses, and pectin, in addition to other minor components.

Although sugar beet pectin has been widely studied for making micro-emulsions [15,16,17], a few studies have reported on the preparation of nano-emulsion [18,19]. To the best of our knowledge, there have been no previous studies regarding use of SBP pectin for making emulsions from PGE.

In addition to the specific properties of pectin isolated from SBP, CNFs with elementary fibrils (width ~5 nm) could be easily isolated from SBP after pectin extraction due to the unique cell wall structure of sugar beet where most of the tissue is parenchymal, which is characterized by only a very thin primary wall and loosely organized cellulose nano-size microfibrils embedded in a matrix of hemicelluloses and pectin [20]. Several publications have studied the isolation of CNFs from SBP using different methods and pretreatments [11].

The well-known unique properties of CNFs such as the high specific surface area, the nanometer wide and micrometer length dimensions, the combination of unique intrinsic mechanical strength with good flexibility properties, and the ability of strong hydrogen bonding along the nanofibers or with other different matrices motivate their application in different areas including papermaking. CNFs can improve mechanical, barrier (air, moisture, oil, and thermal), and printability properties, and reduce paper weight products [21,22,23,24,25,26,27,28]. Nevertheless, due to the negative effect of CNFs on the drainage of water during paper sheet formation as a result of their very small size, very high water holding capacity, and ability to fill empty spaces between the pulp fibers and clog pores of the wet web [27,29,30], applying CNFs as a surface coating after paper sheet formation instead of applying them as a paper additive has resulted in increasing interest for improving paper properties and the production of novel paper products [31,32,33,34,35,36,37,38]. Usually, paper coating materials are used in very small quantities and applied as a very thin layer, yet they bring about significant effects on paper properties. Specifically, CNFs isolated from SBP were successfully used as a coating material for paper sheets to improve mechanical and oil-proof properties, as well as air permeation resistance [31].

While there is no use of pectin from any resources in papermaking so far, its use with CNFs has been reported in alginate scaffolds for biomedical use [39], to improve water resistance of soybean protein [40], as a co-carrier to improve water redispersibility of spray-dried CNFs in water [41], to prepare aerogel with improved mechanical properties [42], and for the preparation of printing inks [43,44].

However, both pectin and CNFs have no antibacterial activity due to their polysaccharide nature. Therefore, their use together in products with antibacterial properties requires the addition of an antibacterial agent. For pectin, adding PGE to it has been used for the preparation of antibacterial edible coatings of fruits [45,46] or in food products such as jam and juice [46,47]; pectin used in the aforementioned studies was isolated from pineapple, orange, pomegranate, and banana. However, there is no previous work published so far on using PGE with SBP pectin for making nanoemulsion or use of their mixture in papermaking.

Based on the above-mentioned limitations of pectin and CNFs for use in antibacterial paper products, the current manuscript studies the preparation of nanocomposite from pectin/PGE nanoemulsion and CNFs for use as a bio-based and environmentally friendly films and coating for different applications such as food packaging materials.

In fact, one of the most appealing solutions and modern approaches for preventing microbial or virus contamination and their spreading is through designing appropriate surface coating, i.e., fabrication of antimicrobial or antivirus surfaces [48]. This can be achieved through adding a specific antibacterial or antivirus agent, such as pomegranate extract, in a coating matrix; this is known as an active antimicrobial or antivirus coating approach. Pomegranate extract contains different small molecules with antimicrobial and antivirus activities which work with different mechanisms to de-activate viruses and bacteria [2,3,4,49,50,51,52,53,54,55].

In the current work, CNFs were used as the film forming material to impart strength, pectin was the emulsifying agent to form nanoemulsions from pomegranate extract to make it compatible with CNFs, and pomegranate extract was the antibacterial agent.

## 2. Materials and Methods

### 2.1. Raw Material and Reagents

Wet-pressed SBP (~20 wt.% solid content) was kindly supplied by the Alnubariah Company for Sugar, Alexandria, Egypt. It was directly dried in an oven with hot air circulation at 50 °C for 12 h. The chemical composition of the SBP was 38.02% α-cellulose, 18.2% pentosans, 3.85% lignin, 2.77% ash, 19.40% galacturonic acid content, and 10.13% protein content, as determined according to standard methods of chemical analysis [56,57,58].

Sodium chlorite (technical grade 80%), glacial acetic acid, sulfuric acid, sodium thiosulfate, potassium bromide, potassium bromate, hydrochloric acid, acetic acid, citric acid, and sodium hydroxide were of analytical grade. They were purchased from Fisher Scientific U.K. Ldt (Loughborough, UK) and used as received. Polyamideamine-epichlorohydrin (PAE) crosslinking agent was commercial grade (solid content ~33 wt.%, Solines, Wilmington, DE, USA). PAE solution was diluted to 1 wt.% with distilled water before use. *E. coli* EMCCN 3060 and *S. aureus* EMMCN 3057 bacteria were kindly supplied from the Egyptian Microbial Culture Collection Network (EMCCN) at the National Research Centre, Giza, Egypt.

### 2.2. Extraction of Pectin

Extraction of pectin by acid hydrolysis was carried out as previously published by Abou-Elseoud et al., as follows [59]: SBP was suspended in water at a liquor ratio of 1:15 and acidified to pH 1 with sulfuric acid. It was then heated for 2 h at 85 °C under mechanical stirring. The residue was then separated from the soluble compounds by vacuum filtration, washed with distilled water, and kept in the fridge at 4 °C until use for isolation of the nanofibers.

To isolate pectin from the filtrate, it was centrifuged in 50-mL centrifuge tubes at 10,000 rpm for 10 min to remove fines, and pectin was precipitated by the addition of ethanol at volume ratio of 3:1 ethanol to filtrate; the mixture was left for 2 h and the precipitated pectin was centrifuged at 10,000 rpm for 20 min, washed with 70% ethanol/water mixture, centrifuged again, and dried at 40 °C for 48 h. The chemical composition of the extracted pectin was 72.6% galacturonic acid, 10.64% neutral sugars, 10.5% protein, and 0.5% ferulic acid [59].

### 2.3. Isolation of Cellulose Nanofibers (CNFs)

Isolation of CNFs from the de-pectinated SBP was carried out according to the previously published protocol [31]. In brief, after bleaching with acetic acid/chlorite mixture, the bleached de-pectinated SBP was suspended in water at 2 wt.% consistency and subjected to high shear mixing using ESR-500x laboratory high shear homogenizer (ELE, Shanghai, China) at 10,000 rpm for 15 min. The suspension was then passed twice through a two-chamber Homolab 2.2 high-pressure homogenizer (FBF, Parma, Italy). The CNF suspension was then kept in fridge at 4 °C until use. The chemical composition of isolated CNFs was 87.8% α-cellulose, 6.96% pentosans, 0.45% lignin, 1.10% ash, 2.44% galacturonic acid content, and 0.31% protein content, as determined according to standard methods of chemical analyses [56,57,58].

A JEM-2100 high-resolution transmission electron microscope (HRTEM) (JEOL, Tokyo, Japan) was used for characterizing the microstructure of the isolated CNFs after being stained with phosphotungstic acid solution.

### 2.4. Extraction and Characterization of Pomegranate Extract (PGE)

Pomegranate peels were washed with water, oven-dried in an oven with hot air circulation at 40 °C for 24 h, and then ground to pass through a 20-µm sieve. The extraction was carried out using 70/30 (v/v) ethanol/water mixture in a Soxhlet for 8 h at 85 °C. The solvent containing the extract was then evaporated at 65 °C using a Rotavapor R-210 rotary evaporator (BÜCHI Labortechnik AG, Flawil, Switzerland) under vacuum. To complete the drying, the highly viscous extract was poured in a glass Petri dish and dried in an oven under vacuum for 18 h at 65 °C. Serial dilution was made from the extract and kept for further analysis.

### 2.5. Determination of Individual and Total Phenolics

The Folin–Ciocalteu assay, adapted from Ramful et al. [60], was used for determination of total phenolics. In addition, the individual phenolic compounds were determined by high-performance liquid chromatography (HPLC) using an Agilent 1260 series (Agilent, Santa Clara, CA, USA). The separation was carried out using a C18 column (4.6 mm × 250 mm i.d., 5 μm). The mobile phase consisted of water (A) and 0.02% tri-fluoro-acetic acid in acetonitrile (B) at a flow rate 1 mL/min. The mobile phase was programmed consecutively in a linear gradient as follow: 0–5 min (80% A); 0–5 min (80% A); 5–8 min (40% A); 8–12 min (50% A); 12–14 min (80% A); and 14–16 min (80% A). The multi-wavelength detector was monitored at 280 nm. The injection volume was 10 μL for each of the sample solutions. The column temperature was maintained at 35 °C.

### 2.6. DPPH Radical Scavenging Activity

The effect of extracts on 1,1-diphenyl-2-picrylhydrazyl (DPPH) free radical was estimated according to the procedure described by Aboelsoued et al. [61]. The absorbance was measured at 517 nm using Jenway 7305 UV-visible spectrophotometer (Jenway, Staffordshire, England). The control was conducted with ethanol instead of the sample. DPPH scavenging capacity was calculated by using the following equation:

$$Scavenging\;activity\;\left(\%\right)=\frac{Ac-As}{Ac}\times 100$$

where *Ac* and *As* are the absorbance at 517 nm of the control and sample, respectively.

L-ascorbic acid solutions as standards were also analyzed by DPPH and ABTS methods. The total antioxidant values of citrus samples were expressed as mg g^–1^ dry weight L-ascorbic acid equivalent antioxidant capacity (VCEAC).

### 2.7. Determination of Ferric Reducing Power (FRAP) Assay

The FRAP assay is based on the ability of phenolics to reduce Fe^3+^ to Fe^2+^ [62]. To prepare the FRAP reagent, 0.1 M acetate buffer (pH 3.6), 10 mM TPTZ, and 20 mM ferric chloride (10:01:01, v/v/v) were mixed. Then, 20 µL of previously diluted extract were added to 150 µL of reagent. The absorbance was measured at 593 nm using Jenway 7305 UV-visible spectrophotometer (Jenway, Staffordshire, England).. The analysis was performed in triplicate, using an aqueous Trolox solution as standard, and the results were expressed as lmoles Trolox equivalents/100 g of fresh weight sample.

### 2.8. Preparation and Characterization of Pectin/Pomegranate Extract (Pectin/PGE) Emulsion

Pectin/PGE emulsions containing 2.5, 5, 10, 15, and 20 wt.% of PGE (based on the oven dry weight of pectin) were prepared by ultrasonic treatment under cooling at 4 °C in an ice bath for 2 min using a UP 400 Hielscher ultrasonic processor (Hielscher Ultrasonics GmbH, Teltow, Germany); A 1-cm diameter probe was used at an amplitude of 75%. The concentration of Pectin/PGE in water was 4.5%.

TEM of the emulsions was carried out using JEM-2100 HRTEM (JEOL, Tokyo, Japan) for characterizing the microstructure of the emulsion after being stained with phosphotungstic acid solution.

The antimicrobial activity of the prepared Pectin/PGE emulsions was evaluated by minimal inhibitory concentration test (MIC) as previously described [63]. Gram-positive bacteria (*Staphylococcus aureus*) and Gram-negative bacteria (*Escherichia coli*) were used as test organisms. A pre-culture of bacteria was grown in Tryptic Soy Broth medium overnight at 37 °C and a serial dilution were made from each strain until obtained dilutions of 1 × 10^5^ and 1 × 10^8^ CFU/mL for *Staphylococcus aureus* and *Escherichia coli*, respectively. Next, 100 µL of both tested bacteria were added to the test tubes containing 9.9 mL of sterile Tryptic Soy Broth medium and exposed to 0.1 g of the different Pectin/PGE emulsions, which were oven-dried at 65 °C under vacuum; a neat pectin sample was tested as a blank. All samples were then incubated at 37 °C with shaking at 140 rpm for 24 h. After a 24 h incubation, a series of dilutions were prepared by the addition of 1 mL of each culture to 9 mL of sterile 0.3 mM phosphate buffer (pH 6.8), followed by seeding 100 µL of each culture onto an agar plate. The plates were incubated at 37 °C for 24 h and the surviving cells counted. The antimicrobial activity was expressed as a reduction of the bacterial colonies after contact with the test specimen and compared to the number of bacterial colonies from the blank sample (neat pectin). The percentage reduction (inhibition) was calculated using the following equation:

% Reduction = ((*B − A)/B*) × 100

where *A* is the surviving cells (CFU—colony forming units) for the plates containing the treated substrate and *B* is the surviving cells from the control.

### 2.9. Preparation and Characterization of CNFs/Pectin/PGE Films

CNFs/Pectin/PGE films containing different ratios of Pectin/20%PGE emulsion were prepared by casting suspension mixtures in a 9-cm diameter Teflon petri dish. Glycerol was added at a fixed ratio of 25% of all samples to get good film formation without damage due to shrinkage. The ratios of Pectin/20%PGE were 2.5, 5, 7.5, 10, 15, and 20 wt.% of the oven dry weight of CNFs plus glycerol. Then, 2% of PAE wet strength agent (based on total weight of the films) was added to all samples. The suspensions were dried at 40 °C for 18 h in an oven with hot-air circulating. The produced films were conditioned at 50% relative humidity for 48 h at 25 °C before testing.

Tensile strength properties were measured using LR10 K Lloyd instrument (Lloyd Instruments, Fareham, UK) with a 1 kN load cell at 25 °C using a crosshead speed of 2 mm/min. Strips with 10 × 90 mm width by length, respectively, were used and the distance between the grips was 20 mm. Five specimens from each sample were measured and the results averaged.

Anti-bacterial activity of the films was tested using the disk diffusion method [63]; Gram-positive *Staphylococcus aureus* and Gram-negative *Escherichia coli* bacteria were used as test organisms. A loopful from each stock strain was transferred into 10 mL of Tryptic Soy Broth medium with 0.6% yeast extract and incubated at 37 °C overnight. Then, 100 µL from each strain was seeded on the surface of Tryptic Soy agar plates, and ~1-cm^2^ of the films was placed onto the inoculated surfaces and then incubated at 37 °C for 24 h to detect the bacterial inhibition zones. The experiment was performed in triplicate.

### 2.10. Coating of Paper Sheet

CNFs/20%Pectin/PGE suspension with 3% solid content containing 2% of PAE (based on total dry weight of the suspension) was used for coating commercial wrapping paper sheets, which have a basis weight of ~30 g/m^2^. The paper sheets were fixed over a glass plate and coated with the suspension using ZUA 2000 coater (Zehntner GmbHTesting Instruments, Sissach, Switzerland), coating was carried out using a gap of 500 µm.

Coated paper sheets were dried in air circulation oven at 80 °C for 15 min for crosslinking of PAE. The coated paper samples were conditioned at 50% relative humidity for 48 h at 25 °C before testing. The amount of CNFs/Pectin/PGE coating was determined gravimetrically as g/m^2^ from the difference in basis weight of coated and uncoated paper sheets, as follows:

$$\begin{array}{c} {\text{Amount of coating (g/m }}^{2}\text{)}=\\ {\text{Basis weight of coated paper sheet in g/m}}^{2}-{\text{Basis weight of uncoated paper sheet in g/m}}^{2} \end{array}$$

### 2.11. Characterization of Paper Sheets

The surface and cross-section of the coated paper was examined by scanning electron microscopy (SEM) using an FEI Quanta 200 scanning electron microscope (FEI Company BV, Eindhoven, The Netherlands) with an acceleration voltage of 20 kV. Paper sheet samples were coated with gold using a sputter coater system (Edwards Sputter Coater, Sussex, UK) before testing.

Tensile strength testing of paper sheets was carried out using LR10K Lloyd universal testing machine (Lloyd Instruments, Fareham, UK) with a 1 KN load cell at a constant crosshead speed of 2 mm/min according to TAPPI T494 (TAPPI 2006). Porosity was measured using a Gurley air permeability tester 4110 (W. & L.E. Gurley, Troy, NY, USA) according to ASTM D726-58. A greaseproof test was carried out using turpentine oil (TAPPI standard T454). A water vapor permeability test was carried out according to the ASTM standard (ASTM E96); all the tests were performed in triplicate at an atmospheric pressure (1 atm) and the results were averaged. WVP was calculated according to the following equation:

WVP (g·m^−1^s^−1^Pa^−1^) = (*m*·*e*)/(*A*·*t*·*p*)

where *m* is the mass increase (in g) of the CaCl_2_, *A* is the area of the film, and *t* is the exposure time in the chamber. The thickness of the film is *e* and *p* is the partial water vapor pressure difference across both of the film specimens corresponding to 0–60% RH, i.e., 1875 Pa.

The overall migration test was carried out according to EU Regulation Nr. 10/2011. Three stimulants were used in the test which represents water, fatty, and acidic conditions: 10% v/v ethanol in water, 50% v/v ethanol in water, and 3% acetic acid solution. All samples were compared to a blank water sample (Millipore water with resistivity 18.5 MΩ) as a reference. As the single sided cell method was used, results were calculated considering the area of only one surface of the test specimen. After 10 days at 40 °C, the samples were picked up from contact, and the extraction aqueous solutions were collected in flasks, heated in a rotary evaporator under vacuum to dryness, and weighed until constant weight (EN 1186-5-single side contact in cell test) [64].

### 2.12. Statistical Analysis

The results of tested samples were presented as the mean ± SD using Microsoft Excel. For the total phenolics and antioxidant activity testing, the data were statistically analyzed by one-way ANOVA using SPSS software version 20. Significance was considered at a level of 0.05.

## 3. Results and Discussion

### 3.1. Composition of PGE

The components of the ethanol/water PGE were characterized by HPLC. Table 1 shows the components separated by the HPLC column and their percentages; the HPLC chromatogram is attached in the Supplementary Information Figure S1. The major components in the PGE are in accordance with previous studies on PGE extracted using ethanol solutions [65].

### 3.2. Pectin/PGE Emulsions

Using PGE extracted with ethanol with hydrophilic polymers, such as cellulose, requires emulsification with suitable emulsifier, which acts as a compatibilizer between the relatively hydrophobic groups of PGE and the hydrophilic hydroxyl groups at the cellulose surface.

#### 3.2.1. Particle Size of Pectin/PGE Emulsions

**Figure 1:**
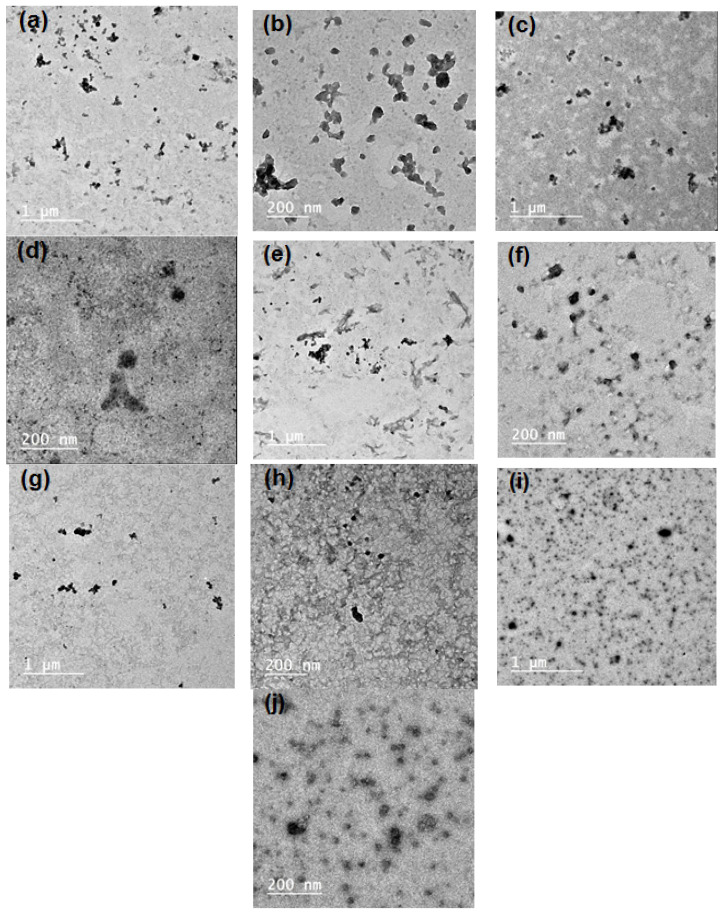
Transmission electron microscopy TEM images of the prepared emulsions from Pectin and pomegranate extract (PGE) at different magnifications: (**a**,**b**) Pectin/2.5% PGE, (**c**,**d**) Pectin/5% PGE, (**d**,**e**) Pectin 10% PGE, (**f**,**g**) Pectin/15% PGE, and (**h**,**i**) Pectin/20% PGE.

**Figure 2:**
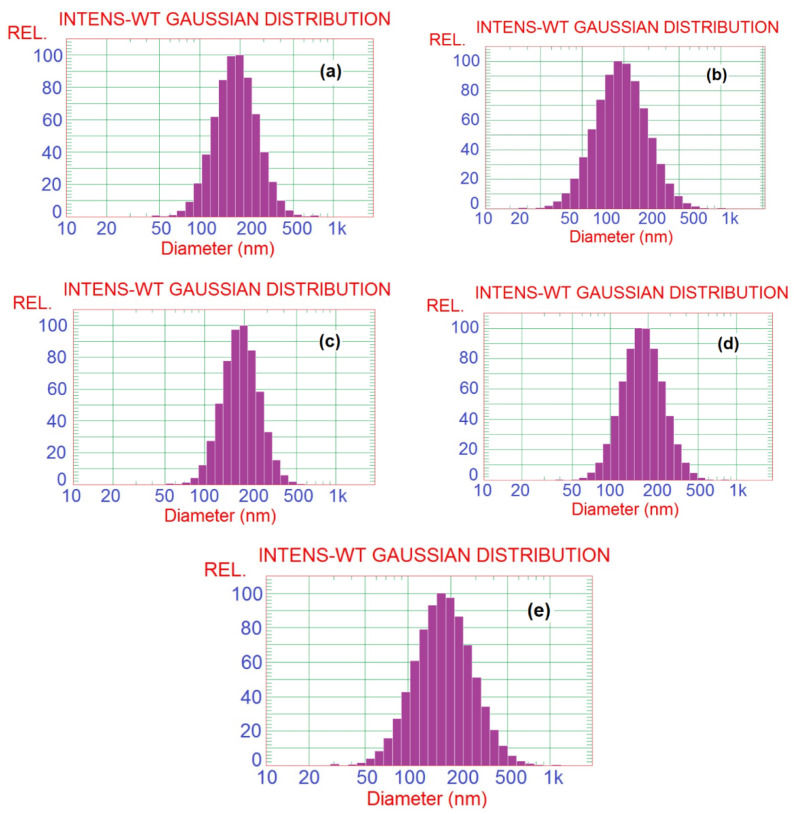
Particle size analysis histograms of Pectin/PGE emulsions with different PGE loadings: (**a**) 2.5% PGE, (**b**) 5% PGE, (**c**) 10% PGE, (**d**) 15% PGE, and (**e**) 20% PGE.

SBP pectin is characterized by a high ability to form micro- and nano-emulsion thanks to the presence of high content of acetyl groups at its polysaccharide chains [11]. In the current work, pectin could form nanoemulsions from the PGE, as shown in the TEM images in Figure 1. The size of the separate emulsion particles was in the range from ~25 to 50 nm for the emulsions with different PGE loading, i.e., the size emulsion particles was not dependent on the concentration of PGE used. It was also noticed that the nanoparticles form several aggregates, but their size was still in the submicron range and less than ~200 nm. Results of particle size analysis by a laser in Figure 2 and Table 2 show a similar trend of the results, e.g., emulsion particles size not dependent on their concentration in pectin, with diameters in the range from 193 to 208 nm. This reflects the high ability of pectin to form emulsions in the nano-diameter range, even at a high content of PGE (20%). The higher mean diameter results in the case of particle size analysis than that in the case of using TEM could be due to the different principals of measuring instruments used, and the formation of aggregates from the nanoemulsion particles. Cumulative particle size analysis in Table 2 also shows that 90% of the particles had diameters less than ~296, 330, 284, 292, and 323 nm for pectin emulsions containing 2.5, 5, 10, 15, and 20% of PGE, respectively, again indicating the ability of sugar beet pectin to emulsify high ratios of PGE.

#### 3.2.2. Antibacterial Activity of Pectin/PGE Emulsion

Since pectin is a polysaccharide without antibacterial activity, one of the aims of the current work was preparing pectin emulsion with antibacterial activity for different applications. PGE extract is known to have strong antibacterial properties against Gram-positive and Gram-negative bacteria thanks to the presence of several polyphenolic constituents in the PGE, including chlorogenic acid, catechins, and gallic and ellagic acid [66,67]. Several mechanisms for the antibacterial activity of PGE were proposed, including the binding of the polyphenols to the bacterial protein [49,50,51], destroying the synthesis of bacterial RNA and DNA [52,53], and destabilization of the outer bacterial membrane [54,55]. In the current study, the antibacterial activity of the PGE/pectin emulsions was studied to ensure the potential use of the prepared nanoemulsion as an antimicrobial mixture and that the emulsification did not affect the antimicrobial activity of PGE.

Table 3 shows the % inhibition of Pectin/PGE nanoemulsion with different PGE loadings. As seen in the table, even at the lowest PGE concentration used, ~83 and 80% inhibition against *E. coli* and *S. aureus* could be achieved, respectively. There was no increase in the % inhibition at PGE loading >15%, where % inhibition was ~92% and 99.5% against *E. coli* and *S. aureus*, respectively. The observed higher activity of PGE extract against the *S. aureus* than that of *E. coli* is in agreement with the results of previous studies [5,58,59,60,61,62,63,64,65,66,67,68].

#### 3.2.3. Antioxidant Activity of Pectin/PGE Emulsion

As mentioned above, PGE is rich in polyphenolic compounds which give it a strong antioxidant and antibacterial activity. The total phenolics (such as gallic acid), antioxidant assay by the DPPH (2,2-diphenyl-1-picryl-hydrazyl hydrate) free radical method, and Trolox equivalent’s antioxidant capacity (TPTZ) of PGE, Pectin, and Pectin/PGE nanoemulsion were determined, and the results are shown in Table 4.

As shown in the table, PGE had a high content of total phenolics, which is in accordance with the previous finding of Aboelsoued et al. [61]. On the other hand, pectin extracted from sugar beet had a significant amount of phenolics, reaching 10.07 mg gallic acid/g of pectin (*p* < 0.05). The Pectin/PGE nanoemulsion also showed antioxidant activity but the value of total phenolics was low since PGE represents 20% of the dry weight of Pectin/PGE, and also the emulsion had a concentration of 4.5% in water. The antioxidant activity was measured using both DPPH as mg ascorbic acid per gram sample and TPTZ as µg Trolox eq/g sample methods, in accordance to screening the activities of samples. PGE had the highest DPPH radical scavenging activity (785.23 ±1.94 mg ascorbic acid per gram sample, *p* < 0.05). On the other hand, pectin and Pectin/PGE nanoemulsion had DPPH antioxidant activities of 4.34 ± 0.01 and 3.49 ± 0.03 mg ascorbic acid per gram, respectively. The reducing power activity method TPTZ (FRAP) showed the same trend as the DPPH results. Xiong et al. [69] suggested that the antioxidant activity of pectin could be due to the higher content of electrophilic groups, which could accelerate the release of hydrogen from OH groups of the different sugars in pectin and act as an electron donor.

HPLC analysis results of Pectin/PGE in Table 5 were in accordance with antioxidants and total phenolics results mentioned above. Supplementary Information Figure S2 shows the chromatogram of Pectin/PGE nanoemulsion. Some of the phenolics in PGE could not be detected in the HPLC chromatogram of the Pectin/PGE nanoemulsion due to their originally low concentration in PGE. In addition, some phenolics such as syringic acid, ferulic acid, apigenin, and hesperetin phenolics appeared in the Pectin/PGE nanoemulsion chromatogram, which could have originated from pectin.

### 3.3. CNFs/Pectin/PGE films

**Figure 3:**
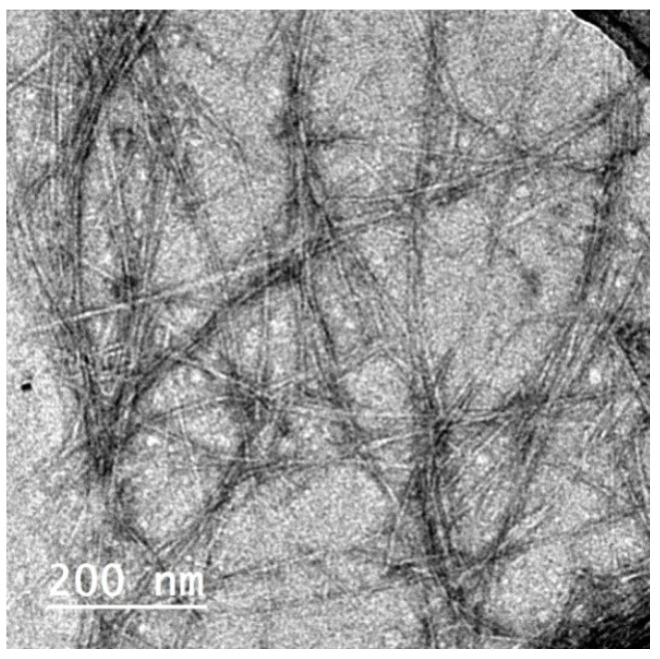
TEM image of cellulose nanofibers (CNFs) isolated from de-pectinated sugar beet pulp (SBP).

CNFs isolated from the de-pectinated SBP were used for making films with Pectin/PGE emulsion; CNFs had ~5 nm thickness and were several microns in length, as shown in the TEM image in Figure 3 [31]. The Pectin/PGE emulsion chosen for mixing with CNFs was that containing 20% of PGE since it gave the highest antibacterial activity toward the studied Gram-positive and Gram-negative bacteria. CNF films containing different ratios of the chosen Pectin/PGE emulsion were prepared. The ratios of the Pectin/PGE emulsion ranged from 2.5% to 20% based on the dry weight of the CNFs and the fixed ratio of PAE (2 wt.% based on the weight of the total film) was added. Films with good formation and homogeneity were obtained. It should be pointed out that glycerol was added to the films as a plasticizer in order to study the effect of Pectin/PGE emulsion, because without the addition of glycerol, the films suffered from significant shrinkage and cracking.

#### 3.3.1. Tensile strength properties of CNFs/Pectin/PGE films

**Figure 4:**
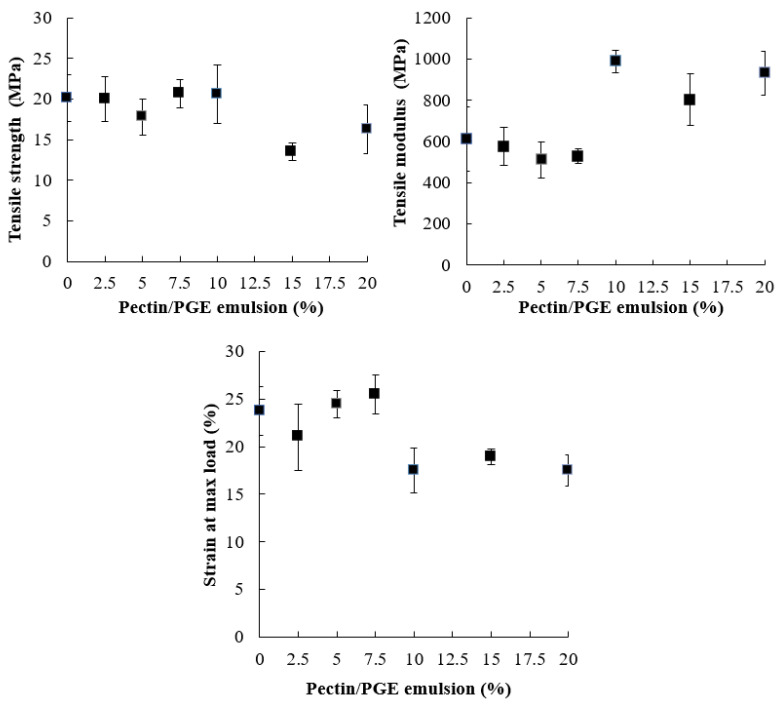
Tensile strength properties of CNFs/Pectin/PGE films with different loadings of PGE.

The effect of Pectin/PGE on the tensile strength properties of CNFs is shown in Figure 4. Tensile strength of cellulose fiber films is controlled by fiber-to-fiber shear strength, fiber tensile strength, and fiber pull-out work from the cellulose sheet [70]. Fiber-to-fiber bonding has a more positive effect than the length of fibers [71]. As Figure 4 shows, the presence of Pectin/PGE in the CNFs matrix did not affect its tensile strength up to the addition of 10% of the former. This indicates that the presence of Pectin/PGE particles acted as links between CNFs through hydrogen bonding and compensated the expected loss in tensile strength due to decreasing the CNF content. Pectin contains carboxylic groups which are highly polar and their presence in the films could induce stronger hydrogen bonding that improves the shear strength of fiber–fiber bonds [72]. However, at Pectin/PGE loadings of 15 and 20%, the tensile strength of the films decreased by about 19 to 32% compared to blank CNF film. This means that, at high Pectin/PGE loadings (15–20%), the fiber-to-fiber shear strength significantly decreased and exceeded the effect of hydrogen bonding between pectin and CNFs, and finally lead to the decreasing tensile strength of the films.

Regarding Young’s modulus of the films, it was not affected by Pectin/PGE addition until addition of 10% was reached, i.e., the films became stiffer with an addition ≥10% of Pectin/PGE. The strain at the maximum load also showed a significant decrease at Pectin/PGE loadings ≥10%.

#### 3.3.2. Greaseproof properties of CNFs/Pectin/PGE Films

An important requirement for films used in the packaging of oily or fatty products is the greaseproof property. Polymers rich in hydrophilic groups, such as cellulose and pectin, can form an extensive network of hydrogen bonding and have very low interaction with oil and grease [36]. In addition, CNF films have a very dense structure which, along with extensive hydrogen bonding, makes the penetration of oils and fats rather difficult [36]. In the current work, the grease resistance of CNF films containing different ratios of Pectin/PGE nanoemulsion was studied to investigate the effect of the presence of the nanoemulsion particles on that property. As shown in Table 6, all films showed good greaseproof property, especially with the addition of ≥ 5% of Pectin/PGE nanoemulsion. According to the standard method used, time more than 30 min for the permeation of oil across the films qualifies them as high greaseproof material. The remarkable increase in greaseproof property as a result of increasing the Pectin/PGE content could be attributed to the presence of more pectin, which contains carboxylic groups originating from the galacturonic building units in its structure (~72 wt.% of pectin is galacturonic acid). The carboxylic groups have higher water affinity than hydroxyl groups due to higher polarity of the formers. When the nanoemulsion of Pectin/PGE formed, the polar groups of pectin are directed outward, and thus the outer surface of the nanoemulsion particles is highly polar.

#### 3.3.3. Antibacterial Activity of CNFs/Pectin/PGE Films

**Figure 5:**
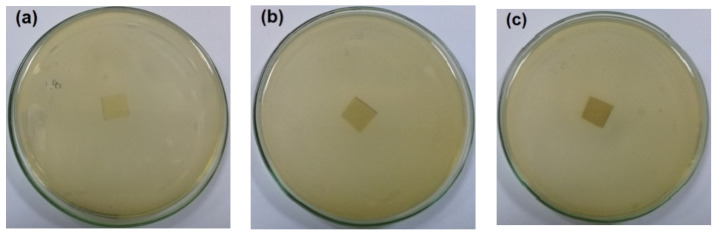

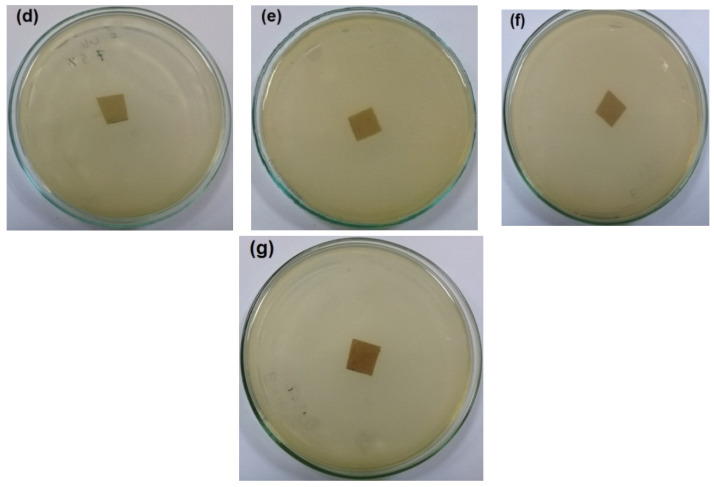
Images of the antibacterial test of CNFs/Pectin/PGE films against *S. aureus*: (**a**) blank CNFs, (**b**) CNFs + 2.5% Pectin/PGE, (**c**) CNFs + 5% Pectin/PGE, (**d**) CNFs + 7.5% Pectin/PGE, (**e**) CNFs + 10% Pectin/PGE, (**f**) CNFs + 10% Pectin PGE, and (**g**) CNFs + 20% Pectin/PGE.

**Figure 6:**
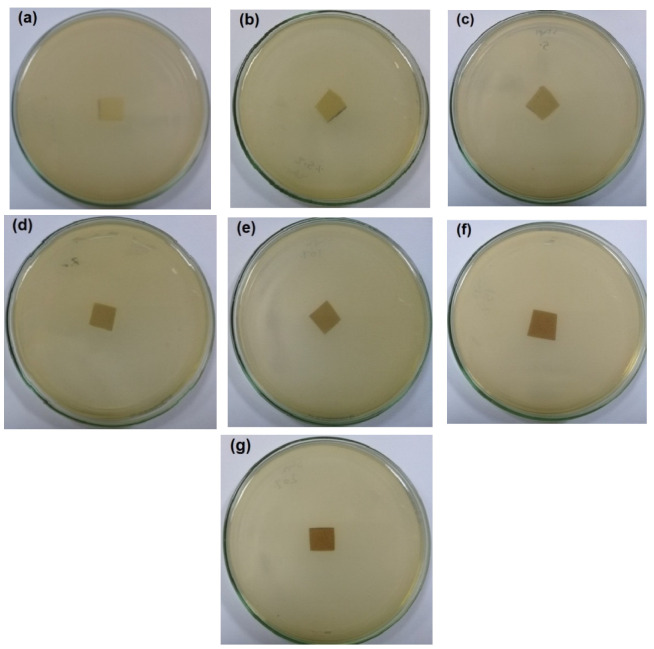
Images of the antibacterial test of CNFs/Pectin/PGE films against *E. coli*: (**a**) blank CNFs, (**b**) CNFs + 2.5% Pectin/PGE, (**c**) CNFs + 5% Pectin/PGE, (**d**) CNFs + 7.5% Pectin/PGE, (**e**) CNFs + 10% Pectin/PGE, (**f**) CNFs + 15% Pectin PGE, and (**g**) CNFs + 20% Pectin/PGE.

The antibacterial activity of CNFs/Pectin/PGE containing different ratios of Pectin/PGE emulsions was studied in order to obtain films with appropriate antibacterial properties. The disc diffusion technique, which is a widely used quantitative method for studying antimicrobial properties of films, was used against *S. aureus and E. coli* bacteria, which are the most popular bacteria contaminants in foodstuff. The images of the test are shown in Figure 5 and Figure 6. The method measures the diameter of the non-infected zone (so called the inhibition zone) in mm around the sample due to the release of the antibacterial agent from the sample to the agar media used in the test. As the images in the figures show, blank film made from CNFs and crosslinking agent did not show any antibacterial activity, and spreading of the *E. coli* and *S. aureus* bacteria took place on the film. In the case of testing CNFs/Pectin/PGE films against *S. aureus*, films with Pectin/PGE content from 2.5 to 5% showed the spread of the bacterium in the whole Petri dish and on the surface of the films, while that containing ≥7.5% Pectin/PGE exhibited a clear surface and the bacterium could not grow on the film. In the case of testing the films against *E. coli*, a bacterium-clear surface of the films could be seen for the sample containing ≥10% of Pectin/PGE.

It should be pointed out here that the films did not show a clear inhibition zone around them in the test. In our study, since the antibacterial agent (PGE) was emulsified with pectin and both are embedded in the CNF matrix, PGE components could not release in highly sufficient amounts from the film to the surroundings during the test, and therefore no clear inhibition zone formed. However, there was no growth of the studied bacteria on the surface of the films, i.e., an antibacterial surface could be obtained.

#### 3.3.4. Total Phenolics and Antioxidant Activity of CNFs/Pectin/PGE Film

Table 7 shows total phenolics and antioxidant activity of CNFs/20% pectin/PGE film; data of those of pectin and Pectin/PGE nanoemulsion are also added to the table. It is interesting to see that CNF film had antioxidant activity and a small amount of total phenolics. This could be due to the attachment of residual pectin to the isolated CNFs [33]. CNFs/20% Pectin/PGE film showed antioxidant activity but, as expected, it was lower than that of Pectin/PEG nanoemulsion since the film contains 20% of Pectin/PEG.

### 3.4. Paper Sheets Coated with CNFs/Pectin/PGE Emulsion

Coating of paper is an important industrial application since it can impart new properties on paper products using only small amounts of coating materials, which are usually more expensive than blank paper sheets. Using CNFs for paper coating is very interesting from both a scientific and industrial point of view due to the unique properties of the thin layer of CNFs formed at the paper surface. In fact, coating using CNFs is more preferred than the addition of CNFs to pulp before the making of paper sheets due to the negative effect of CNFs on paper sheet formation and drying because of the very high water affinity of CNFs, possible loss through the sieve of the machine, and also the lack of homogenous distribution in the final paper sheets [36].

**Figure 7:**
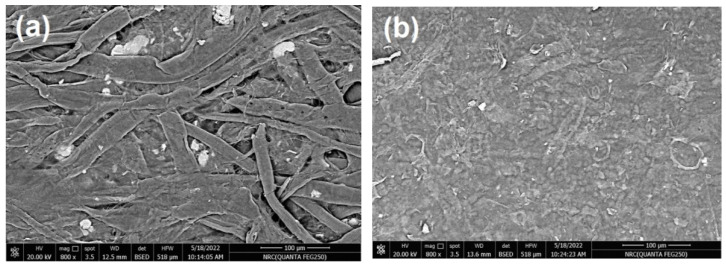

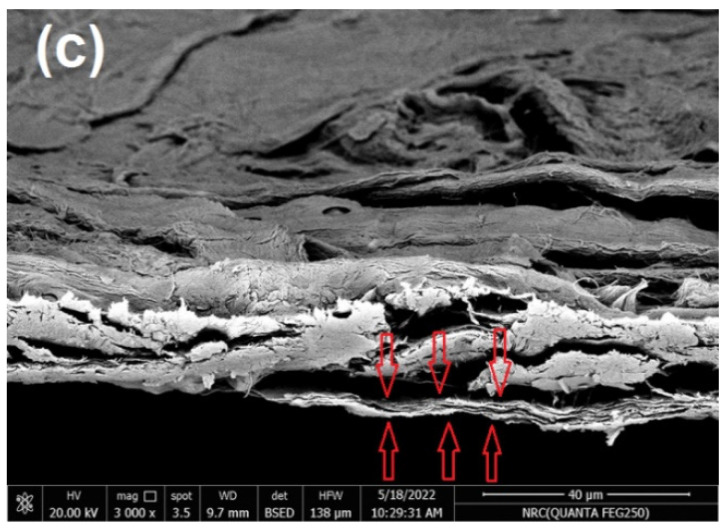
Scanning electron microscopy (SEM) of (**a**) the surface of a paper sheet before coating, (**b**) the surface of a paper sheet after coating with CNFs/20% Pectin/PGE, and (**c**) the cross-section of a paper sheet after coating with CNFs/20% Pectin/PGE.

In the current work, commercial paper sheets used for wrapping were coated with CNFs/20% Pectin/PGE mixture since this formulation gave good antibacterial properties, as seen in the previous section. The paper sheets used had a thickness of ~0.04 mm and a basis weight of ~30 g/m^2^. The coating layer of the applied CNFs/20% Pectin/PGE had a basis weight of ~5.8 g/m^2^; it was selected after a preliminary trial to obtain good and homogenous coverage of paper sheets. SEM images of paper sheets before and after coating are shown in Figure 7; the images showed good coverage of the paper sheets with the dense plastic-like layer of the CNFs/Pectin/PGE; thickness of the coated layer was about 4 µm, as shown from the cross section image (Figure 7c).

#### 3.4.1. Mechanical Properties of Coated Paper Sheets

The effect of coating paper sheets with CNFs/20% Pectin/PGE on tensile strength properties was studied, and the results are shown in Table 8. As the results show, coating of paper sheets with the small thickness layer of CNFs/20% Pectin/PGE resulted in a slight decrease (~7.5%) in tensile strength in the machine direction (MD) of paper sheets, but no significant change occurred in the cross direction (CD). The Young’s modulus of coated paper sheets decreased in both CD and MD; the decrease was about 28% and 33% in the MD and CD of paper sheets, respectively, which means that the paper sheets became more stretchable. This was clear from the increase in strain at the maximum load of paper sheets in both the MD and CD directions. The decrease in tensile strength properties in spite of applying the CNFs/20% Pectin/PGE layer could be attributed to the effect of wetting and drying paper sheets during coating [73].

In addition to testing tensile strength properties of the coated paper sheet in the dry conditions, the wet tensile strength of paper sheets with and without coating was studied using the ASTM method where folded strips were subjected to tensile force when immersed in water. The results showed that the wet tensile strength of a blank paper sheet was 3.26 ± 0.14 and 1.70 ± 0.11 N in the MD and CD directions of paper sheets, respectively while that of coated paper sheets was 3.53 ± 0.53 and 2.13 ± 0.24 N in the MD and CD directions of paper sheets, respectively. These results mean an increase in the wet tensile strength of paper sheet of about 8.3 and 25% in the MD and CD directions, respectively, by the very thin layer of CNFs/20% Pectin/PGE.

The improved wet tensile strength of coated paper sheets could be attributed to the formation of a tight network of crosslinked CNFs at the surface of paper sheets which can reduce the penetration of water. Previous results showed the significantly lower water absorption of sheets made from CNFs than that from the pulp fibers [25,74]. In addition to the aforementioned reason, the addition of a crosslinking agent to the CNFs/Pectin/PGE mixture could also result in the crosslinking of the fibers of the paper sheet, which thus became more resistant to water.

#### 3.4.2. Physical Properties of Coated Paper Sheets

In spite of the small thickness of applied coating with CNFs, all previous work showed its effectiveness in changing properties of paper sheets and also imparting them with new properties [31]. In the current work, the effect of the CNFs/20% Pectin/PGE coating on air porosity, water vapor permeability, and grease resistance of paper sheets was studied; the results are shown in Table 9. Regarding air porosity, due to the very tight structure of the CNFs/20% Pectin/PGE film formed on the surface of paper sheets, the porosity was significantly decreased, and the time required to pass 100 mL of air increased by about 7 times. Such a decrease in air porosity is also responsible for the high barrier properties of CNFs films for gases including oxygen [75,76,77]. It is interesting to see that air porosity of the paper sheets coated with CNFs/20% Pectin/PGE is higher than that obtained in a previous work using only a neat CNF coating with about the same thickness [31]. In that study, the time required for passing 100 mL of air across a paper sheet with a 4-µm CNF coating (~4.5 g/m^2^) was about 160 s. This, in fact, means that the presence of pectin/PGE emulsion between the CNFs network exerted more of a barrier for the passing of air.

Regarding the moisture barrier property (water vapor permeability) of paper coated with CNFs/20% Pectin/PGE, the situation is different because of the high hydrophilic nature of CNFs which work in an opposite direction to the effect of a very tight structure of CNF coating. In humid conditions, CNF swelling increases the tendency for the permeability of moisture [78]. The presence of Pectin/PGE emulsion, where the hydrophilic functional groups of the emulsified nanoparticles are to the outside and encapsulating the hydrophobic PGE, could also increase the tendency for water vapor permeability. As a result of the aforementioned two opposite effects, the net result found in the current work is that there was no significant effect of the CNFs/20% Pectin/PGE coating on water vapor permeability of paper sheets. A similar effect was found before in the case of coating paper sheets with neat CNFs [31].

Regarding the grease resistance of CNFs/20% Pectin/PGE coated paper, it was found that the coating significantly improved the grease resistance of paper sheets but not to the level that meets the required standards. In fact, the greaseproof property of CNFs originates from the extensive hydrogen bonding network, which result in high cohesive energy density and low interaction between CNFs and grease [36,79].

#### 3.4.3. Migration Testing

Coated paper used as packaging materials should have stability of the coated layer, especially if they are in contact with aqueous, fat, or acidic products. In the case of adding functional additives to the coating layer, their release upon contact with different stimulants could be beneficial. In the current work, testing the stability of the CNFs/20% Pectin/PGE layer coated on paper sheets was carried out in 10% alcohol/water, 50% alcohol/water, and 3% acetic acid/water solutions, which represent aqueous, fat, and acidic stimulants; the test was carried out at 40 °C for 10 days, which represent the conditions for long-term storage at or below room temperature, including 15 min of heating up to 100 °C or 70 °C for up to 2 h [64]. Paper sheets coated with CNFs only were also tested as controls. As the results in Table 10 show, blank paper sheets showed constant weight loss upon their contact with the different stimulants (~1.8–2 mg/dm^2^). According to the standard method used, this loss is lower than the maximum limit allowed for the migration of coated material (<10 mg/dm^2^). On the other hand, paper sheets coated with CNFs/20% Pectin/PGE showed more weight loss as a result of immersion in the different stimulants. The highest weight loss was in aqueous stimulant (3.46 mg/dm^2^), while the lowest loss was in the fat stimulant (2.3 mg/dm^2^); acidic stimulant resulted in a loss of 2.95 mg/dm^2^. Loss of Pectin/PGE with their antimicrobial and antioxidant activities as a result of contact with the different stimulants could be beneficial in packaging applications. The small values of the weight loss indicate the slow release and these very little amounts of Pectin/PGE could migrate into the food in contact with the coated paper. These very small amounts will not affect the color, taste, and properties of the food. In addition, the antibacterial properties, along with the antioxidant ones, could lead to increasing the shelf life of the food product. Testing of the prepared coated paper in packaging of different food products is planned to be studied in future work.

## 4. Conclusions

With the aim to prepare films and coating materials with antioxidant and antibacterial activities from renewable bio-based resources, CNFs/Pectin/PGE nanocomposites were successfully prepared. Pectin and CNFs were isolated from sugar beet pulp, and pomegranate extract (PGE) was extracted from pomegranate peels. The isolated sugar beet pectin could be used as an emulsifier to PGE to prepare nanoemulsions with antimicrobial and antioxidant activities, as well as good compatibility with CNFs. The size of the Pectin/PGE nanoemulsion particles was not dependent on the concentration of PGE, while the antimicrobial activity at the different PGE loadings (from 2.5 to 20%) ranged from 83 to 92% and from 80 to 99.7% against *E. coli* and *S. aureus*, respectively. Homogenous films with antimicrobial and antioxidant activity, as well as flexibility, good mechanical properties, and high greaseproof properties could be prepared. Presence of Pectin/PGE nanoemulsion particles in CNF films improved their greaseproof property thanks to the hydrophilic outer surface of the nanoemulsion. The surface of the CNFs/Pectin/PGE films acquired antibacterial properties against Gram-positive and Gram-negative bacteria. However, because PGE was emulsified in pectin and the Pectin/PGE was embedded within the crosslinked CNFs network, the release of the pectin/PGE was in very small amounts. This could be beneficial for the packaging of food products since the very slow release means an insignificant effect on the packaged food regarding its quality properties such as color or taste, and at the same time, could extend the shelf life by the antimicrobial effect.

In addition to the films prepared, the CNFs/Pectin/PGE mixture could be successfully used as a coating mixture for the commercial wrapping of paper sheets. The micrometer-scale coating (~4 µm) applied to paper sheets could impart them with a high barrier to air and improved greaseproof property, without affecting the water vapor permeability of paper sheets. The applied CNFs/Pectin/PGE layer can increase the wet tensile strength of paper sheets, i.e., higher stability in wet conditions, but did not improve these properties in dry conditions. The coated CNFs/Pectin/PGE film showed good adhesion to the paper sheet surface in different solutions representing aqueous, alcoholic, and acidic media at 40 °C for 10 days. Based on the obtained results, it could be concluded that antimicrobial and antioxidant films and coating for wrapping paper could be prepared and used safely in contact with different food products, extending the shelf life as well as keeping good quality attributes.

## Figures and Tables

**Table 1:** Composition of pomegranate extract (PGE)obtained by high-performance liquid chromatography (HPLC).

Constituent	Concentration (µg/g)
Chlorogenic acid	21.95
Gallic acid	11.18
Ellagic acid	9.51
Catechin	5.55
Coffeic acid	3.06
Methyl gallate	0.50
Naringenin	0.36
Pyro catechol	0.081
Rutin	0.066
Cinnamic acid	0.042

**Table 2:** Particle size analysis of Pectin/PGE emulsions with different PGE loadings.

	Mean Diameter (nm)	Variance (P.I)	Cumulative Analysis of Particle Size
Pectin/2.5% PGE	198.0 ± 72.3	0.13	25% of distribution < 144.7 nm 50% of distribution < 185.1 nm 75% of distribution < 236.8 nm 80% of distribution < 251.7 nm 90% of distribution < 295.6 nm 99% of distribution < 432.8 nm
Pectin/5% PGE	208.7 ± 89.9	0.186	25% of distribution < 142.1 nm 50% of distribution < 190.0 nm 75% of distribution < 254.2 nm 80% of distribution < 273.1 nm 90% of distribution < 330.2 nm 99% of distribution < 518.0 nm
Pectin/7.5% PGE	198.1 ± 64.0	0.104	25% of distribution < 151.2 nm 50% of distribution < 187.9 nm 75% of distribution < 233.7 nm 80% of distribution < 246.7 nm 90% of distribution < 284.3 nm 99% of distribution < 398.4 nm
Pectin/10% PGE	193.2 ± 73.2	0.144	25% of distribution < 139.2 nm 50% of distribution < 179.7 nm 75% of distribution < 232.0 nm 80% of distribution < 247.2 nm 90% of distribution < 292.0 nm 99% of distribution < 433.9 nm
Pectin/20% PGE	197.9 ± 92.4	0.218	25% of distribution < 129.4 nm 50% of distribution < 177.4 nm 75% of distribution < 243.0 nm 80% of distribution < 262.8 nm 90% of distribution < 322.7 nm 99% of distribution < 525.6 nm

**Table 3:** Inhibition of *E. coli* and *S. aureus* bacteria by Pectin/PGE emulsion with different PGE loadings.

	% Inhibition	
Sample	*S. aureus*	*E. coli*
Pectin + 2.5% PGE	80.0 ± 4.38	83.3 ± 7.07
Pectin + 5% PGE	84.6 ± 4.35	83.3 ± 7.07
Pectin + 10% PGE	95.4 ± 4.35	86.7 ± 4.71
Pectin + 15% PGE	99.2 ± 0.22	91.7 ± 2.36
Pectin + 20% PGE	99.7 ± 0.22	91.7 ± 4.71

**Table 4:** Total phenolics and antioxidant activity profile of PGE, Pectin, and Pectin/PEG emulsion.

Samples	DPPH Antioxidant Activity (mg Vitamin C/g Sample)	TPTZ (µg Trolox eq/g Sample)	Total Phenolic Compounds (mg Gallic Acid eq/g Sample)
PGE	785.23 ± 1.94 ^c^	3387.6 ± 35.55 ^c^	88.65 ± 4.91 ^f^
Pectin	3.49 ± 0.03 ^b^	93.19 ± 0.01 ^b^	10.07 ± 0.12 ^c^
Pectin/PGE emulsion	4.34 ± 0.01 ^b^	100.9 ± 0.28 ^b^	9.79 ± 2.13 ^e^

Means in each column with different letters are significantly different (*p* < 0.05) ± SD. Analysis performed using ANOVA and the Duncan test.

**Table 5:** Phenolic compounds of PGE and Pectin/PGE emulsion determined by high-performance liquid chromatography (HPLC).

Phenolic Compounds	PGE mg/g	Pectin/PGE Emulsion mg/mL
Gallic acid	11.18	0.06
Chlorogenic acid	21.95	0.012
Catechin	5.55	0
Methyl gallate	0.50	0.001
Coffeic acid	3.06	0.008
Syringic acid	0	0.003
Pyro catechol	0.081	0
Rutin	0.066	0.002
Ellagic acid	9.51	0
Ferulic acid	0.000	0.003
Naringenin	0.36	0
Querectin	0.000	0.002
Cinnamic acid	0.042	0
Apigenin	0	0.001
Hesperetin	0	0.002

**Table 6:** Greaseproof results of CNFs/Pectin/PGE films.

Sample	Film Thickness (mm)	Time for Oil Penetration through Paper Cross Section (min)
CNFs	0.166 ± 0.004	14 ± 2.0
CNFs/2.5% Pectin/PGE	0.186 ± 0.009	16 ± 1.8
CNFs/5% Pectin/PGE	0.164 ± 0.008	˃45
CNFs/7.5% Pectin/PGE	0.189 ± 0.009	˃45
CNFs/10% Pectin/PGE	0.186 ± 0.008	˃45
CNFs/15% Pectin/PGE	0.174 ± 0.007	˃45
CNFs/20% Pectin/PGE	0.188 ± 0.007	˃45

**Table 7:** Total phenolics and antioxidant activity profile of CNFs film, Pectin/PEG emulsion, and CNFs/Pectin/PGE film.

Samples	DPPH Antioxidant Activity (mg Vitamin C/g Sample)	TPTZ (µg Trolox eq/g Sample)	Total Phenolic Compounds (mg Gallic Acid eq/g Sample)
CNFs/Pectin/PGE film	1.60 ± 0.02 ^a^	10.93 ± 0.2 ^a^	4.75 ± 0.53 ^b^
CNFs film	1.97 ± 0.04 ^a^	8.81 ± 0.08 ^a^	2.55 ± 0.12 ^a^
Pectin/PGE emulsion	4.34 ± 0.01 ^b^	100.9 ± 0.28 ^b^	9.79 ± 2.13 ^e^

Means in each column with different letters are significantly different (*p* < 0.05) ± SD. Analysis performed using ANOVA and the Duncan test.

**Table 8:** Tensile strength properties of blank and paper sheets coated with CNFs/20% Pectin/PGE mixture.

Sample	Tensile Strength (MPa)		Young’s Modulus (GPa)		Strain at Max. Load (%)	
	MD *	CD *	MD *	CD *	MD *	CD *
Blank paper sheets	28.14 ± 1.58	15.99 ± 1.98	5.91 ± 0.61	3.99 ± 0.24	1.53 ± 0.25	1.30 ± 0.17
CNFs/Pectin/PGE coated paper sheets	26.02 ± 1.22	15.35 ± 1.48	4.21 ± 0.19	2.69 ± 0.26	1.65 ± 0.23	1.70 ± 0.19

* CD and MD are the cross and machine direction of paper sheets, respectively.

**Table 9:** Effect of CNFs/20% Pectin/PGE coating on the physical properties of paper sheets.

Sample	Porosity (s/100 mL)	Water Vapor Permeability (gm^−1^s^−1^Pa^−1^) × 10^−11^	Grease Resistance (Time for Oil Sorption through Paper Cross Section in Minutes)
Blank paper sheets	36 ± 2	2.15 ± 0.091	Immediate sorption across paper thickness
CNFs/Pectin/PGE coated paper sheets	280 ± 8	2.10 ± 0.19	2.8 ± 0.31

**Table 10:** Overall migration test results of CNFs/20% Pectin/PGE coated paper sheets.

Sample	Weight Loss (mg/dm^2^) upon Contact with Stimulant		
	10% Alcohol	50% Alcohol	3% Acetic acid
Blank paper sheets	1.960 ± 0.02	1.87 ± 0.05	1.83 ± 0.07
CNFs/Pectin/PGE coated paper sheets	3.46 ± 0.03	2.31 ± 0.05	2..95 ± 0.06

## Data Availability

All data are presented in the manuscript.

## Supplementary Materials

The following supporting information can be downloaded at: https://www.mdpi.com/article/10.3390/polym14214605/s1, Figure S1: High-performance liquid chromatography (HPLC) chromatogram of pomegranate extract (PGE); Figure S2. High-performance liquid chromatography (HPLC) chromatogram of Pectin/PGE emulsion.
