# Supplementary material for: Cellulose Nanofibers/Pectin/Pomegranate Extract Nanocomposite as Antibacterial and Antioxidant Films and Coating for Paper
Source: Polymers (Basel). 2022 Oct 30;14(21):4605. doi: 10.3390/polym14214605 (PMC9659057; doi:10.3390/polym14214605)
Supplement: Supplementary file 1 [file polymers-14-04605-s001.zip › polymers-2000977-supplementary.pdf]

Supplementary information file for:

Cellulose Nanofibers/Pectin/Pomegranate Extract Nanocomposite as Antibacterial and Antioxidant Films and Coating for Paper

Enas Hassan [1], Shaimaa Fadel [1], Wafaa Abou-Elseoud [1,2], Marwa Mahmoud [3] and Mohammad Hassan [1,2,*]

[1] Cellulose and Paper Department, National Research Centre, 33 El-Buhouth Street, Dokki, Giza 12622, Egypt
[2] Advanced Materials and Nanotechnology Group, Centre of Excellence for Advanced Sciences, National Research Centre, 33 El-Buhouth Street, Dokki, Giza 12622, Egypt
[3] Food Technology Department, National Research Centre, 33 El-Buhouth Street, Dokki, Giza 12622, Egypt
* Correspondence: ml.hassan@nrc.sci.eg

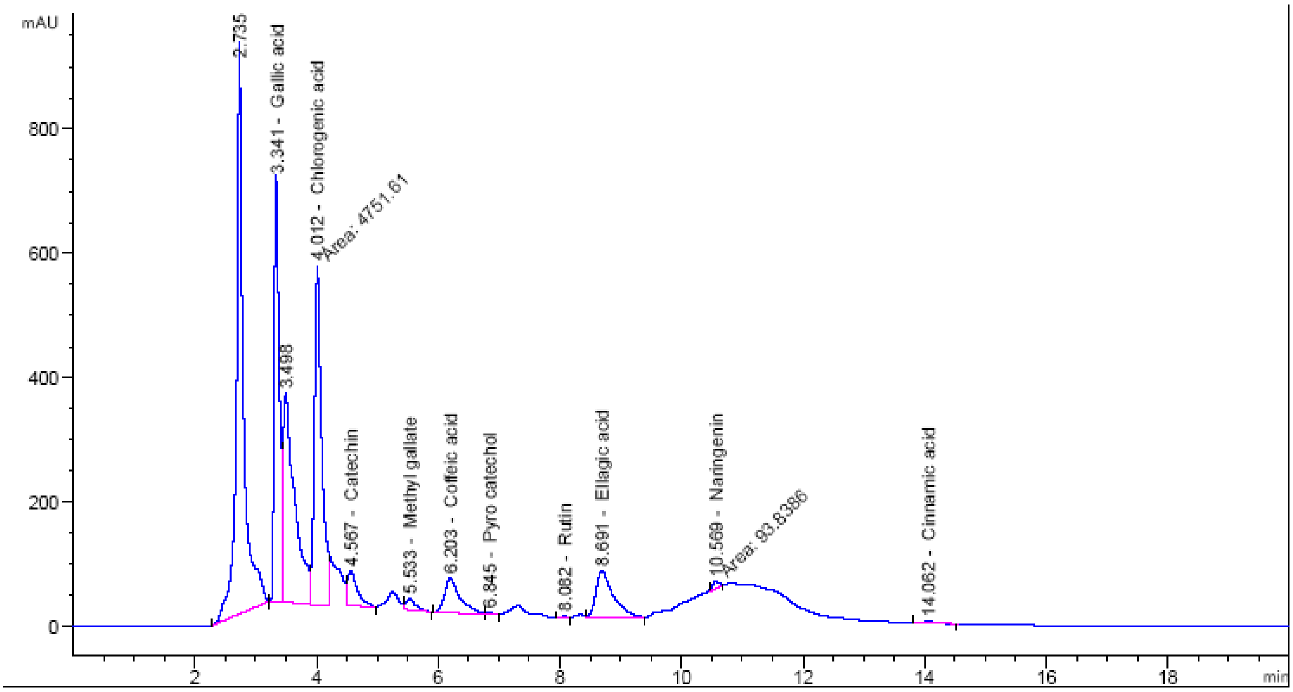

Figure S1. High-performance liquid chromatography (HPLC) chromatogram of pomegranate extract (PGE).

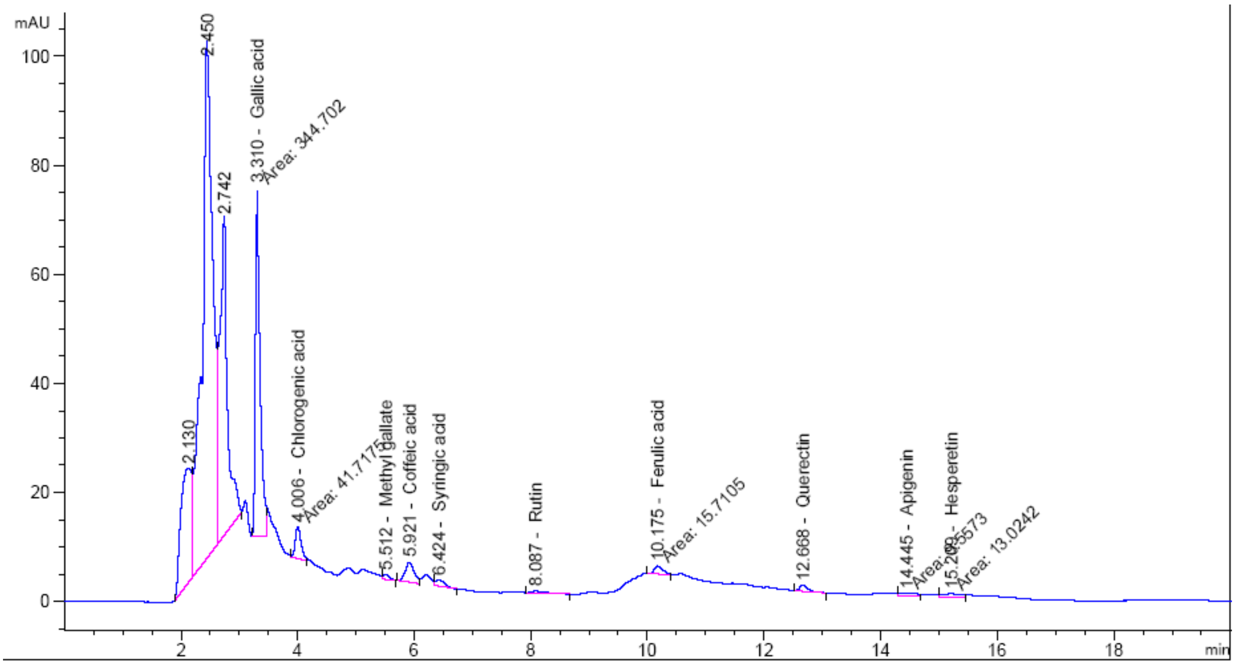

Figure S2. High-performance liquid chromatography (HPLC) chromatogram of Pectin/PGE emulsion.
